# Supplementary material for: Wide range of G6PD activities found among ethnic groups of the Chittagong Hill Tracts, Bangladesh
Source: PLoS Negl Trop Dis. 2020 Sep 14;14(9):e0008697. doi: 10.1371/journal.pntd.0008697 (PMC7514097; doi:10.1371/journal.pntd.0008697)
Supplement: S1 Table — (DOCX) [file pntd.0008697.s001.docx]

| **Assay** | **Primers** | **Oligonucleotides** |
| --- | --- | --- |
| Mahidol | MAHIDOL_F | 5ˊ-GCGTCTGAATGATGCAGCTCTGAT-3ˊ |
|  | MAHIDOL_R | 5ˊ-CTCCACGATGATGCGGTTCAAGC-3ˊ |
| Viangchan | VIANGCHAN_F | 5ˊ-CCTGAGGGCTGCACATCT-3ˊ |
|  | VIANGCHAN_R | 5ˊ-GTCGTCCAGGTACCCTTTGGGG-3ˊ |
| Mediterranean | MEDIT_F | 5ˊ-ACTCCCCGAAGAGGGGTTCAAGG-3ˊ |
|  | MEDIT_R | 5ˊ-CCAGCCTCCCAGGAGAGAGGAAG-3ˊ |
| Orissa | ORISSA_F | 5ˊ-CAGCCACTTCTAACCACACACCT-3ˊ |
|  | ORISSA_R | 5ˊ-CCGAAGTTGGCCATGCTGGG-3ˊ |
| Kalyan-Kerala | VIANGCHAN_F | 5ˊ-CCTGAGGGCTGCACATCT-3ˊ |
|  | EX10R | 5'- GATCACCAGCTCGTTGCGCTTG -3' |
