# Supplementary material for: Wide range of G6PD activities found among ethnic groups of the Chittagong Hill Tracts, Bangladesh
Source: PLoS Negl Trop Dis. 2020 Sep 14;14(9):e0008697. doi: 10.1371/journal.pntd.0008697 (PMC7514097; doi:10.1371/journal.pntd.0008697)
Supplement: S2 Table — *Optimized temperature. (DOCX) [file pntd.0008697.s002.docx]

| **Assay** | **Annealing (°C)** | **PCR Product**  **(bp)** | **Restriction**  **Enzyme** |
| --- | --- | --- | --- |
| Mahidol | 58.0°C | 104 | *Hind* III |
| Viangchan | 64.0°C | ̴ 200 | *Hpy* 188III |
| Mediterranean* | 63.0°C | 545 | *Mbo*II |
| Orissa* | 60.6°C | 345 | *Hae*III |
| Kalyan-Kerala* | 63.0°C | ̴ 540 |  |
