# Supplementary material for: Wide range of G6PD activities found among ethnic groups of the Chittagong Hill Tracts, Bangladesh
Source: PLoS Negl Trop Dis. 2020 Sep 14;14(9):e0008697. doi: 10.1371/journal.pntd.0008697 (PMC7514097; doi:10.1371/journal.pntd.0008697)
Supplement: S3 Table — (DOCX) [file pntd.0008697.s003.docx]

| **Variable** | **Coefficient β (95%CI)** | **p** |
| --- | --- | --- |
| Mahidol hemizygous / homozygous | -0.478 (-0.542 to -0.413) | <0.001 |
| Mahidol heterozygous | -0.208 (-0.283 to -0.133) | <0.001 |
| Orissa hemizygous / homozygous | -0.338 (-0.525 to -0.151) | <0.001 |
| Hemoglobin concentration | -0.140 (-0.150 to -0.130) | <0.001 |
| Chakma ethnicity | 0.083 (0.019 to 0.147) | 0.011 |
| Chak ethnicity | -0.115 (-0.182 to -0.049) | 0.001 |
| Bengali ethnicity | -0.065 (-0.129 to -0.000) | 0.050 |
| Sex – females | -0.087 ((-0.119 to -0.055) | <0.001 |
| Weight | 0.004 (0.003 to 0.005) | <0.001 |
