# Supplementary material for: Wide range of G6PD activities found among ethnic groups of the Chittagong Hill Tracts, Bangladesh
Source: PLoS Negl Trop Dis. 2020 Sep 14;14(9):e0008697. doi: 10.1371/journal.pntd.0008697 (PMC7514097; doi:10.1371/journal.pntd.0008697)
Supplement: Attachment — Submitted filename: Ley et al_PLoSNTD_Response to Reviewers_v0.3.docx [file pntd.0008697.s007.docx]

Dr Wuelton Monteiro

Associated Editor

*PLoS NTD*

24^th^ June 2020

**Re.** PNTD-D-20-00793

*"Wide range of G6PD activities found among ethnic groups of the Chittagong Hill Tracts, Bangladesh"*

Dear Dr Monteiro,

Thank you for your email regarding our manuscript and the opportunity to revise our submission. The reviews were favourable and acknowledged the importance of this study and the clinical implications of our findings, while raising a number of issues regarding the presentation and limitations of the study.

We have now addressed all of the reviewers’ and editorial comments, making revisions to the manuscript to clarify our methodology and its caveats, and highlight the implications of our findings.

We believe that the revised manuscript is much improved for these changes and has important clinical implications for the radical cure of malaria that will be vital for malaria elimination.

We hope that you would agree that the paper is now suitable for publication.

Thank you for your time and consideration.

Yours sincerely,

Benedikt Ley on behalf of the other authors

**Editorial Comments:**

**Assessment of phenotypes for G6PD is really valuable. Unfortunately, the quality of the data as presented so far adds limitations to the manuscript. The limitations need to be discussed to avoid these, findings might be then generalized and used inappropriately in the future. To be even more clear, the idea that there is not good correlation between phenotypes and genotypes in G6PD is most often caused by poor laboratory technique or poor sample handling. Technical limitations of the manuscript need to be highlighted. I recommend to present full data set in an universal format such as csv.**

We agree with the editor that the technical challenges of G6PD phenotyping may have confounded our findings. However, we have confidence in our collaborators at icddr,b, who have more than 20 years experience in this field and are the only WHO accredited laboratory for the evaluation of G6PD diagnostics. The laboratory has also been accredited by PATH (USA) for G6PD related laboratory work. However limitations with G6PD phenotyping are inherent and we have now revised the manuscript considerably to address these concerns. We have also included a full csv dataset to provide greater transparency to the analysis.

***All line numbers refer to the clean version of the resubmitted manuscript***

**Reviewer #1:**

1. **“… Hill Tracts (CHT) carry the highest malaria burden …” better say people living in the Hill tracts?**

We have revised the text as requested:*“The population of the multi-ethnic Chittagong Hill Tracts (CHT) carry the highest malaria burden within Bangladesh.”* (line 31 – 32)

2. **Table 1: the column “mean body temp. …” is not helpful and could be omitted or replaced with number of febrile participants detected?”. Same is true for the sentence “The mean axillary body temperature was 36.3°C (95% confidence interval (95%CI): 36.2 – 224 36.3) …”**

We have added a column to table 1 as requested. The text already contains the number of febrile participants: “*The mean axillary body temperature was 36.3°C (95% confidence interval (95%CI): 36.2 – 36.3) and 0.4% of all participants (4/1002) had a temperature above 37.5°C (table 1*).” (line 233 – 234)

3. **A figure illustrating the assembly of the participants (consort chart) could be useful?**

We now include the consort chart as figure 1.

4. **The regression model is difficult to understand. Please revise the Table 5 so as to show the affected/non affected number of participants for each row in a separate column?**

We have expanded on the multivariate regression model in the methods section, where it now reads: “*Multiple regression analysis was performed to identify key variables affecting non-normalized G6PD activity. Non-normalized G6PD activity (in U/dL) was selected as dependent variable since very low Hb levels may affect G6PD activity…*” (lines 197 – 200).

We have also rephrased the respective paragraph in the results section: “*Significant independent variables in order of contribution that predicted G6PD activity were genotype (except for the Kalyan Kerala variant), Hb concentration, ethnicity, sex and weight. Malaria diagnosis, irrespective of method, was not a significant covariate (supplementary table 3).*” (lines 316 – 319)

Following comments from reviewer 1 and 3 we have moved table 5 to the supplement (supplement table 3). The table shows the β-coefficient of all significant independent variables and the corresponding p-value for each variable that predicts G6PD activity as a continuous variable.

5. **There is something missing in the sentence: “…****almost 10% of the population of the had G6PD activities of less than 30%...”**

The sentence has now been corrected.

*“…our results revealed that almost 10% of the population had G6PD activities of less than 30%...”* (line 323 – 324)

6. **The authors speculate regarding the low number of disease/symptom presented by G6PD deficient individuals. “Despite a high proportion of the population being at risk of drug induced hemolysis, there are few reports of drug induced hemolytic reactions from local medical staff (B. Ley, personal communication) and the literature”. This requires more explanations. How are these deficient individuals challenged? Do they receive routinely PQ treatment or other triggers for haemolysis? In the absence of information on exposure to triggers for haemolysis it is difficult to assess the outcome.**

Most malaria in Bangladesh occurs in the Chittagong Hill Tracts, with an increasing proportion of *P. vivax* patients. National treatment guidelines recommend primaquine (PQ) based radical cure for *P. vivax*, and this can cause drug induced hemolysis in G6PD deficient patients. Despite a high prevalence of G6PD deficiency, anecdotally there have been very few reports on PQ induced hemolysis reported in this area.

We have revised the introduction to emphasise these issues and also expanded the discussion on the possible reasons for these contradictory observations.

The respective sentence now reads: *“Despite a high prevalence of G6PD deficiency and thus associated risk of drug induced hemolysis, there are few reports of PQ induced hemolytic reactions either from the local medical staff (C.S. Phru, personal communication) or in the literature (8, 18). The reasons for this are unclear, but could reflect low prescription of PQ by healthcare providers, underreporting of adverse events due to limited access to healthcare, poor treatment adherence to a prolonged course of PQ, or G6PDd individuals having partial protection against malaria (15, 44, 45).”* (line 331 – 337)

7. **I am not convinced by the conclusion that testing could/should be conducted according to ethnicity as even in tribes with low G6PD def. prevalence deficient individuals were detected. It may be wiser to recommend routine use of rapid tests/biosensors for wider use of 8-aminoquinoline regimens?**

We have clarified the conclusion, as follows:*“Whilst testing should ideally be available to identify all individuals with G6PD deficiency, in practice this is often confounded by logistical constraints. Our study highlights that priority should be given for ethnic groups known to have a high prevalence of severe and intermediate G6PD deficiency.”* (lines 366 – 369)

8. **The authors show the mean Hb values for each population in Table 1; however, this data should be presented and analyzed stratified by gender since it differs between men and women.**

We now state this in the text as follows: *“Hb and body temperature differed significantly between ethnic groups, and this was also apparent after stratifying by sex (all p<0.001).*” (lines 239 – 240)

9. **It is not clear why authors included the two individuals of Lushai ethnic group in the analysis since only two subjects are not representative. I suggest removing them and consider analyzing 10 ethnic groups.**

We have rephrased the text, naming only ten ethnicities. The two Lushai participants were bonus and provide the only known record of G6PD in this population so we would like to include these as well. The respective section now reads: *“Between 13th August 2015 and 11th January 2016 a total of 1000 participants from 10 ethnic groups were enrolled into the study. In addition, two participants from the Lushai ethnic group were enrolled, bringing the total sample size to 1002.” (lines 221 – 223).*

In table 1 and table 2 the Lushai are set in italics to distinguish these participants, we have removed Lushai from figure 3.

10. **From the main text or tables I could not find the results for qPCR concerning parasite species. Please add this information to the manuscript.**

The information has now been added to lines (247 - 249): *“Based on PCR a total of 21 participants were positive for P.falciparum, 16 participants were positive for P. vivax and 5 participants harbored both P. falciparum and P. vivax parasites.”*

11. **It is clear from previous studies that heterozygous women cannot be accurately identified through G6PD enzyme activity assays. I suggest presenting G6PD activity data in Table 2 and Fig. 2 stratified by gender. Did the authors find significant differences in G6PD activity among ethnic groups stratified by gender? This could be due to different populations presenting different variants of G6PD gene (for variants assayed and for those that could not be assayed in the present study).**

We found no difference in the sex distribution between the different ethnicities. We have changed the following sentence: *“The proportion of males within each ethnic group ranged from 40.1% to 59.9% and did not differ significantly (p=0.279) (table 1).” (lines 232 -233)*

We have revised figure 3 (previously figure 2) to present data stratified per sex. Table 2 identifies individuals at risk of drug induced hemolysis following primaquine based radical cure, irrespective of sex.

12. **Discussion, the authors claim that malaria status was not found to be a significant predictor of G6PD activity irrespective of diagnostic assay applied. However, it should be made clear that the study was not powered to assess any association between G6PDd and positivity for malaria.**

We have changed the sentence as follows:*“Malaria status was not a significant predictor of G6PD activity irrespective of diagnostic assay applied, however our study was neither designed nor powered to detect this effect .“* (lines 341 – 343).

13. **In Lines 339-342, it was raised the possibility of a recent haemolytic event to explain inconsistent results for genotype-phenotype correlations. But other factors as concomitant haemoglobinopathies and high leukocyte counts could also lead to a false normal G6PD result (Malaria Journal, 2013; 12:391). Did the authors take these other issues into account? Please, discuss these potential factors.**

We agree. We have revised the text to include the recommended reference (#37):*“Although not quantified, study participants may have had recent blood loss due to injury, or substance or pathogen induced hemolysis, thereby replacing older erythrocytes with reduced G6PD activity with younger RBCs with higher activity (54). Neither can we exclude the possibility that concomitant hemoglobinopathies or high leukocyte counts, may have also confounded measured G6PD activity (37).” (lines 349 – 353)*

14. **The authors should evaluate the possibility (due to sample size) of estimating the Hardy-Weinberg equilibrium for each ethnic group.**

We agree that ideally the Hardy-Weinberg equilibrium could be determined per ethnicity and G6PD variant, however the respective numbers are too small for this analysis.

**Minor comments**

15. **Line 161. Please add the reference of qPCR assays used for molecular diagnosis of malaria.**

We have added the respective reference (references 34 and 35)

16. **Lines 166-167. Specify the final concentration of each reagent (or the volume used and the stock concentration) used in the PCR reaction.**

We have added the required information, the respective section now reads: “*P. falciparum varATS qPCR was run in a total volume of 12 µL, containing 0.4 µM of each primer and probe, 6 µL TaqMan FastAdvanced (Applied Biosystems), and 4 µL DNA. P. vivax cox1 qPCR was run in a total volume of 12 µL, containing 0.4 µM of each primer and probe, 6 µL TaqMan FastAdvanced (Applied Biosystems), and 4 µL DNA*.” (lines 163 – 166)

17. **Line 219. This data was not shown in Table 1. This information needs to be removed.**

We have removed this accordingly (line 231)

18. **Line 305. Multivariate is more appropriate than “multivariable” (please see in Am J Public Health., 2013; 103(1): 39–40). While multivariable analysis refers to statistical models in which there are multiple response variables, multivariate analysis refers to statistical models that have ≥ 2 outcome variables.**

We have changed this in line 315

19. **Table 2. I suggest including the number of individuals with no variant determined.**

We have added this to table 2.

20. **Table 2. Correct “Number with with”.**

Revised as suggested.

**Reviewer #2:**

**The manuscript submitted is well written and covers an interesting concept regrading ethnic minorities, G6PDd and targeted screening policies for PQ and TQ. Overall it warrants consideration for publication but requires some clarifications and possible modifications***.*

Many thanks for the review.

**Major Concerns**

1. **For AMM – were your thresholds adjusted for each ethnic group or was it based on overall population? It’s a bit of a catch 22, if you rely on AMM within each subgroup is the population large enough to determine AMM, if you rely on total, does that adequately capture variation at ethnic level – which appears to be a significant driver of U/gHb and a major point of your manuscript. Please clarify what was done. It looks like AMM was determined using population level based on figure 2.**

We calculated the AMM for the entire study population. To clarify this we have rephrased the sentence as follows: *“The adjusted male median (AMM) was calculated and defined as 100% G6PD activity for the entire study population.” (lines 190 – 191).*

The reason for this approach is that a primary objective was to assess whether a population wide testing strategy would be warranted within a multi-ethnic population, and this would not have been possible if we had applied the ethnicity specific AMM.

2. **Spend a little more time in discussion describing how this information could be used to inform policy and what a testing strategy/policy might look like. You touch on this in your conclusion briefly, but you only pay lip service to the issues and complexities of how this information could translate to policy and strategy, when in my opinion, this is the most interesting part of the study.**

We agree that effective implementation of routine G6PD testing is critically important. However, this has been addressed by many other recent reviews on the subject. We reference these reviews and highlight that testing can best be facilitated by knowledge of the ethnic differences in variants and prevalence between populations.

To reflect these considerations, we have included the following sentence to the conclusion: *“Whilst testing should ideally be available to identify all individuals with G6PD deficiency, in practice this is often confounded by logistical constraints. Our study highlights that priority should be given for ethnic groups known to have a high prevalence of severe and intermediate G6PD deficiency. Future research is warranted to identify locally effective strategies to implement routine G6PD testing to guide PQ and TQ based radical cure.” (lines 366 – 370)*

**Minor Comments**

3. **Methods- When was the study performed?**

This is stated at the beginning of the results:*“Between 13^th^ August 2015 and 11^th^ January 2016 a total of 1000 participants from 10 ethnic groups were enrolled into the study.” (Lines 221 -222)*

4. **Line 140 – notice any difference between capillary and venous blood samples?**

We have added the following sentence to address this: “*A total of 11 participants were below the age of 7, the ratio of males to females did not differ between those above and below 7 years of age (p=0.110). When comparing median G6PD activities between both cohorts, no significant difference was observed (p=0.1418)”* (lines 234 – 237)

5. **Citation for line 155?**

We have added this (ref 32; Ley et al. (2017), PLoS One)

6. **Results- Any particular reason you kept Lushai participants? N of 2 adds little value when describing G6PD levels within ethnic group and might lead to confusion for lay audiences. Maybe appropriate to put dashes through their G6PD U/hg and include them in any discussion about genetic variants if you found anything interesting – this isn’t compulsory, but I’d like to hear your rationale.**

This is now clarified at the beginning of the results: *”**Between 13^th^ August 2015 and 11^th^ January 2016 a total of 1000 participants from 10 ethnic groups were enrolled into the study. In addition, two participants from the Lushai ethnic group were enrolled, bringing the total sample size to 1002.” (lines 221 – 223).*

We have highlighted the small sample size by putting the group of Lushai in italics in table 1 and table 2 and have removed Lushai from the former figure 2 which is now figure 3.

7. **Line 219 – * and **table insert doesn’t match what is being described in table**

These have now been removed (table 1).

8. **Line 233 – curious if your negative PCR – positive RDT/Microscopy sample is P. knowlesi. Would your primer set pick this up?**

We have clarified as follows (lines 168 - 169): “*By qPCR, samples were only screened for P. falciparum and P. vivax. Samples were not screened for P. malariae, P. ovale, or P. knowlesi*.*”*

9. **Line 239 – was there any association between time delay for testing and G6PD activity? Any issues with temperature control during sample transport or processing?**

The delay between venepuncture and testing was assessed in a regression analysis, but this was not significant and thus excluded from the final regression model. We have clarified this as follows:*“**Blood samples were stored immediately at 4°C and shipped under controlled conditions to the reference laboratory at the icddr,b in the capital Dhaka for quantitative G6PD measurements…” (lines* 147 – 148).

10. **Table 3. Adjust columns so text fits properly.**

Following the suggestion of reviewer 3 table 3 has now been removed.

11. **Figures- figure resolution could be improved, the inserts by gender are blurry.**

All of the figures have been reviewed and meet publication requirements.

**Housekeeping items**

12. **Line 311 – adjusted R^2 should be 0.5340 not 05340**

The typo has been corrected.

13. **Line 316 awkward sentence, “of the population of the had G6PD…”**

This sentence has been revised as follows:*”…* *our results revealed that almost 10% of the population had G6PD activities of less than 30%,” (line 323 - 324).*

14. **Line 355 awkward use of the word good – “****genotype and phenotype was not good”**

The text has been revised as follows: *“Although G6PD genotype is a key determinant of enzyme activity (46), we observed poor correlation between G6PD genotype and phenotype , a phenomenon that has been described previously (47-50).*” (lines 344 – 345)

15. **General: There is some odd spacing for the manuscript that should be double checked – unless authors submitted in “justified text” setting. Example - comma at end of ln 329 or end of 338 to see what I’m talking about. Also be consistent in single space or double space after period, it looks like it hops around a bit, but again that could be due to use of justified text setting.**

We revised the formatting as requested.

**Reviewer #3**:

**This is an interesting manuscript that describes G6PD genotypes and phenotypes in a malaria endemic area of Bangladesh where potentially hemolytic radical cure for Plasmodium vivax will be used. The aim of the study is reasonable but the laboratory analyses have major issues and some of the results interpretation are questionable. The description of the analyzed population and the sample handling is also not very detailed. Probably my biggest question is: why the authors have used the Hb value assessed in the field (up to 7 days earlier) instead of repeating the Hb measurement the same day as spectrophotometric test? This would have been really easy (with hemocue or CBC) and would have given much more reliable results.**

Many thanks for the review. We have addressed your comment on the Hb measurement further down among the “major issues”.

**Major issues**

1. **Line 123 Can the authors show a map with villages and ethnicities? Was the delay from sample collection to analysis in central lab correlated to village of sampling and/or ethnicity?**

Most of the included villages are very small and not listed as villages on Google Maps, we would have to include a map highlighting an area only. We have instead added a list with village names as supplementary file 1.

For logistical reasons we did not visit all villages for sampling every day during sample collection, but visited villages sequentially, dictated by logistics, security and road conditions. Since villages are more or less mono-ethnic, measured G6PD activities may vary from day to day, however this is likely to reflect differences in the prevalence of ethnicities rather than a systematic error in the laboratory.

We did however include the delay between sample collection and testing in the regression model and found this not to be a significant predictor of G6PD activity.

2. **Line 140 participants <7, how many? Which age range? It is known that G6PD activity is higher in the first 6 months of life, so if small babies were included in the study it should be clearly stated.**

Only children older than 1 years were enrolled. We have now clarified this in lines 119 to 120: *“To ensure a good representation of genetic differences in G6PD alleles, one person per household above the age of one year was selected randomly and invited to participate.”*

3. **Line 155 procedures described earlier need a reference and more info on number of replicates and CV among them, temperature conditions, Quality Controls, accuracy, etc. As far as I have seen in literature, the Randox system does not seem extremely accurate for G6PD activity so the authors might want to give more details to show how it performs in their setting.**

We have added a reference (32) in line 153 and revised the text as follows: *“All procedures followed the manufacturer’s instructions with normal and deficient controls run with every test run. The measurement temperature was set to 37°C, the absorption was read twice, five minutes apart, at 340 nm. G6PD activity was calculated from the difference in absorption between the two measurements, following a formula provided by the manufacturer; no temperature correction factor was applied. A run was repeated in case either control returned a result outside the range recommended by the manufacturer.” (lines 153 – 158)*

4. **Line 156 Hb from the field could be potentially highly unreliable if sample have been shipped without appropriate control of temperature, they could have hemolysed and Hb estimation changes when samples get older (usually after 2-3 days).**

In a previous study we have shown that G6PD activity normalized by a Hb measurement does not change significantly over the course of at least one week if stored at 4°C (Alam et al, PLoS One 2018, DOI: 10.1371/journal.pone.0206331). The median duration between sample collection and G6PD measurement in the laboratory was 2 days (interquartile range: 1-4 days, range 0-7 days). When including the delay between sample collection and measurement in our final multivariate regression analysis, the model remains significant (p<0.001, R^2^=0.5595), however the delay to measurement is not significant (p=0.254), suggesting that the delay between sample collection and measurement did not affect measured G6PD activity or Hb levels. To clarify this we have added the respective reference and have revised the respective paragraph: “*G6PD activity was only measured if samples did not show visible signs of hemolysis, measurement was done on a temperature-controlled Shimadzu UV-1800 spectrophotometer (Kyoto, Japan), using kits and lyophilized controls from Randox Laboratories (Crumlin, UK), following procedures described previously, considering the Hb value measured in the field (32, 33).*” (lines 149 – 153)

5. **Line 192 “The dependent variable was non-normalized G6PD activity (in U/dL), independent variables were backwards selected, initially considering age, gender, body weight, height and temperature, Hb, the delay between sample collection and processing, spectrophotometry result, and G6PD genotype”. Please explain why you used the non-normalized G6PD activity, how gender is independent from G6PD and why body weight and height should be considered variables in this analysis. Please explain why this analysis is even needed.**

We did a multivariate regression analysis to determine what factors predict G6PD activity and to what level. We applied a backwards selection of variables, including all possible variables collected. We subsequently eliminated variables that were not significant.

We chose non-normalized G6PD activity as the dependent variable so we could include Hb concentration as an independent variable (not possible when considering G6PD activity normalized by Hb concentration as dependent variable). We wanted to include Hb concentration, since earlier reports had suggested that very low Hb levels may falsely increase G6PD activity (Winasti Satyagraha et al (2015) PLoS NTD, DOI: 10.1371/journal.pntd.0003602). We found this not to be the case. While Hb was a significant contributor (p<0.001) we observed that G6PD activity would decrease at very small level with increasing Hb levels (Suppl. Table 3).

While G6PD activity can be dependent on sex in case of heterozygous females, this does not preclude the use of this variable in the analysis. Body weight was found to be a small but significant contributor and while we do not offer an explanation why this is the case, we do not feel we should exclude this observation because it does not seem plausible. Height is not included in the final model because it was found to be non-significant.

In order to clarify this, we have revised the text: “*Multiple regression analysis was performed to identify key variables affecting non-normalized G6PD activity. Non-normalized G6PD activity (in U/dL) was selected as dependent variable since very low Hb levels may affect G6PD activity (36)*.” (lines 197 – 200)

6. **Line 203 how were the 10 sample per ethnic group chosen? Where they both females and males? Where they from different villages?**

The 10 samples were selected per ethnic group for genotyping and these were chosen randomly as stated in line 210 to 212: *“Host genotyping was performed on all samples with less than 70% G6PD activity by spectrophotometry as well as 10 randomly selected samples per ethnicity from G6PD normal participants.”*

Since samples were selected randomly, this includes both males and females.

7. **Line 239 The delay between sample collection and analysis is rather large; what is written here does not correspond to what written in the discussion in Line 349.**

Many thanks for pointing out this mistake. We have corrected this in the discussion, where it now reads: *“However, all samples were tested within seven days, the recommended maximum delay for G6PD measurement...” (lines 360 - 361)*

8. **Line 240 “100% G6PD activity was defined as 7.03 U/gHb (interquartile range (IQR): 5.38 – 8.69, total range: 0.73 – 17.17) and median G6PD activity varied significantly with ethnicity (p<0.001).“ Is this the AMM or the Male Median (MM)? The median activity between groups should be compared within the same gender (especially since gender were not represented equally in all groups).**

We have now compared median activities stratified per sex and revised the text as follows:*“100% G6PD activity was defined as 7.03 U/gHb (interquartile range (IQR): 5.38 – 8.69, total range: 0.73 – 17.17) and median G6PD activity varied significantly with ethnicity when considering only males (p<0.001) or females (p<0.001).” (lines 255 – 257)*

9. **Line 249 “when females were included, the Chak had the lowest median G6PD activity (4.92 U/gHb, IQR: 1.83 – 7.12)”; unclear what this means.**

The adjusted male median (AMM) is presented in table 2. However, when calculating the median G6PD activity per ethnicity including females the Chak had the lowest G6PD activity. This is relevant when identifying the sub-population at greatest risk of drug induced hemolysis and is reflected in figure 3.

10. **The AMM in the Chak (Table 2) has a very low first quartile, is this really the AMM or the MM?**

This is the AMM

11. **Line 257-262 and Table 3: these comparisons can only be made if the proportion of females and males in the different groups is the same.**

We have removed table 3, and have revised figure 3, which now shows median G6PD activities stratified by sex and ethnicity.

12. **Line 274: “A total of 133 (13.3%) individuals had intermediate G6PD activity, which was present in 12.2% (49/401) of males and 14.0% (84/598) of females; p=0.465.” The authors need to explain how this is possible. The expected number of males with intermediate activity in a population should be very little. Are these data calculated using the overall AMM or the ethnic specific AMM?**

The objective of this study was to determine the necessity to implement routine G6PD testing within a multi-ethnic population, we therefore applied the overall AMM rather than the ethnicity specific AMM. Since the study population is multi-ethnic with different genotypes present in the population, males hemizygous for a mild G6PD variant may be classified as intermediate G6PD activity within the entire population.

We have revised the text as follows: *“A total of 133 (13.3%) individuals had intermediate G6PD activity based on the population wide AMM, which was present in 12.2% (49/401) of males and 14.0% (84/598) of females; p=0.465.” (lines 284 – 286).*

13. **Line 281-285 “Of the 223 participants diagnosed with G6PD activities below 70%, a G6PD variant was identified in 94 (42.2%) cases, comprising 68/90 (75.6%) G6PD deficient individuals […]. A total of 12 (12.0%) of the randomly selected G6PD normal participants had a detectable G6PD variant.” I see two problems with results here: 1) 75.6% of deficient individuals had none of the analysed mutations. While this is possible, the authors should bring some comparative data on prevalence of other known mutations in the area (not included in this analysis) to give us an idea of how likely it is that they make up >25% of deficient samples. 2) 12% of G6PD normal subjects had a variant, if they are females there is no problem at all, if they are males this needs to be explained.**

We discuss the low rate of confirmed genotypes in the discussion and provide references on earlier reports on genotypes from the area: *“The panel of five G6PD variants that were genotyped was based on earlier reports from the area (8, 26, 53). However, a known G6PD variant was only present in 42% of patients with less than 70% G6PD activity. Given that none of the samples failed PCR, it is likely that the chosen panel did not include all variants present in the region.” (Lines 354 – 357).*

10 of the 12 G6PD normal participants who had an identified G6PD variant were females, we have clarified this accordingly: “*A total of 2 (2.0%) males and 10 females (10%) of the randomly selected G6PD normal participants had a detectable G6PD variant*.” (lines 294 – 295)

14. **Line 288 and Table 4 “In total, 45 males were hemizygous and 12 females homozygous for the Mahidol variant with a median G6PD activity of 0.49 U/gHb (7.0% of the AMM, range: 0.0% to 185.8%)”; same here, based on published data, a hemi or homozygous Mahidol is not expected to have >50% activity, is there any plausible explanation for this result?**

In response to a similar comments from reviewer 2 we have expanded further on this in the discussion, where it now reads: “*Although not quantified, study participants may have had recent blood loss due to injury, or substance or pathogen induced hemolysis, thereby replacing older erythrocytes with reduced G6PD activity with younger RBCs with higher activity (54). Neither can we exclude the possibility that concomitant hemoglobinopathies or high leukocyte counts, may have also confounded measured G6PD activity (37)*.“ (lines 349 – 353)

15. **Line 305-312 I do not see the point of using a regression model and in particular to use the non-normalized G6PD activity.**

Kindly see our reply to comment 5 of reviewer 3.

16. **Line 339 Although this is a possible explanation, I would like the authors to also bring other more likely explanations for this large proportion of genetically deficient but phenotypically normal subjects. As mentioned before, analysing the Hb in the field few days prior the assessment of G6PD activity might have brought a large variation in the G6PD activities. A test such as Hemocue (or even CBC) would have been pretty easy to run in the equipped central laboratory at the same time as G6PD. The delay in G6PD analysis, the way samples were transported (was the temperature checked during transportation? Did any sample show signs of hemolysis?), whether the sample came from small children or adults, accuracy of laboratory test used, village of origin or sampling day of samples with unexpected G6PD phenotypes…all these are factors that need to be clearly stated in the manuscript and taken into consideration when analysing the results.**

We agree that there may be technical issues at play but have addressed the above concerns in the following sections:

1. Delay to testing:
   a. “*The median duration between sample collection and measurement of G6PD activity in the reference laboratory was 2 days (IQR: 0 to 4, range 0 to 7).*” Lines 254 – 255).
   b. Delay to testing is initially included in the multivariate regression model*: “The independent variables were backwards selected, initially considering age, gender, body weight, height and temperature, Hb,* ***the delay between sample collection and processing****, spectrophotometry result, and G6PD genotype.”,* lines 200 - 202. This variable was not found to be a predictor of G6PD activity.
2. Condition of sample: We have clarified this in the methods, which now states: *“G6PD activity was only measured if samples did not show visible signs of hemolysis…”* (lines 149 – 150)
3. Temperature controlled transport of samples: We have clarified this: *“Blood samples were stored immediately at 4°C and shipped under controlled conditions to the reference laboratory at the icddr,b in the capital Dhaka…”* (lines 147 – 148).
4. Age of participants: We have clarified this in the methods, where it now reads: *“**To ensure a good representation of genetic differences in G6PD alleles, one person per household above the age of 1 years was selected randomly and invited to participate.” (lines 119 – 120)*
5. Accuracy of lab procedures: We have clarified the lab procedures and QC procedures in the methods: *“All procedures followed the manufacturer’s instructions with normal and deficient controls run with every test run. The measurement temperature was set to 37°C, the absorption was read twice, five minutes apart, at 340 nm. G6PD activity was calculated from the difference in absorption between the two measurements, following a formula provided by the manufacturer; no temperature correction factor was applied. A run was repeated in case either control returned a result outside the range recommended by the manufacturer.”* (lines 153 – 158)
6. Village of origin: *“Most villages (paras) in the CHT are mono-ethnic”* (line 113), accordingly phenotypic G6PD activity will vary by village cluster, depending on ethnicity.
7. Sampling day: For logistical reasons we did not visit all villages for sampling every day during sample collection, but visited villages sequentially, dictated by logistics, security and road conditions. Since villages are more or less mono-ethnic, measured G6PD activities may vary from day to day, however this is likely to reflect differences in the prevalence of ethnicities rather than a systematic error in the laboratory.
17. **Line 355 “Testing should be considered at least for ethnic groups with high proportions of G6PDd and G6PD intermediate individuals, albeit ethnicity specific testing may be difficult to implement in practice.” Since the data show that all ethnic groups analysed, with the exception of “Tripura”, have a prevalence of G6PD deficiency >5%, it seems that a universal G6PD testing strategy would be more appropriate than an ethnic specific one.**

We have changed the respective sentence, it now reads:  *“Whilst testing should ideally be available to identify all individuals with G6PD deficiency, in practice this is often confounded by logistical constraints. Our study highlights that priority should be given for ethnic groups known to have a high prevalence of severe and intermediate G6PD deficiency. Future research is warranted to identify locally effective strategies to implement routine G6PD testing to guide PQ and TQ based radical cure.” (line 366 – 370)*

**Minor issues/comments**

18. **Line 36 by reading the methods, it is clear that participants where not randomly enrolled. This should be corrected in the whole manuscript.**

The respective sentence in the abstract reads: “*Participants from 11 ethnicities were randomly selected …*” and this reflects our enrolment procedures (line 36). Participants within each ethnicity were selected randomly if written informed consent was provided and participants were at least one year of age. This is clarified in the methods section: *“Each village was mapped using Google Maps (Alphabet Inc., Mountainview, USA) and individual households were identified, numbered and a subset selected at random.*

*To ensure a good representation of genetic differences in G6PD alleles, one person per household above the age of one year was selected randomly and invited to participate.” (lines 116 – 120)*

19. **Line 146 50% discrepancy seems pretty high Line 164 how was the subset chosen?**

The respective sentence reads: *“All slides were read by two independent readers and the mean was recorded, provided that the discrepancy between readings was less than 50% of the smaller value and species identification was in accordance.”* (lines 140 – 142).

These are standard procedures for the reference laboratory at the icddr,b and apply to slides of all six participants that were found to be positive by microscopy.

20. **Line 187 why you test for normality of G6PD activity distribution which is expected to be bimodal?**

We have changed this sentence, it now reads: *“Age was assessed for normality and G6PD activity and age were then compared for significant differences between ethnic groups using the t-test, Mann - Whitney U test or Kruskal Wallis test as appropriate.” (lines 193 – 195)*

21. **Line 214 suggest eliminate the 2 samples from the Lushai ethnic group**

We have rephrased the text, naming only ten ethnicities. Given that we enrolled these two individuals and ran an analysis we would like to include them in the results where relevant. The respective section now reads: “*Between 13th August 2015 and 11th January 2016 a total of 1000 participants from 10 ethnic groups were enrolled into the study. In addition, two participants from the Lushai ethnic group were enrolled, bringing the total sample size to 1002*.” (lines 221 – 223).

In table 1 and table 2 the Lushai are set in italics to distinguish these participants, we have removed Lushai from figure 3.

22. **Line 224 “While Hb and body temperature differed significantly among ethnic groups (all p<0.001)”; was sex and age taken into account?**

We have corrected this, the respective sentence now reads: “*Hb and body temperature differed significantly between ethnic groups, and this was also apparent after stratifying by sex (all p<0.001)*.” (lines 239 – 240)

23. **Line 325-334 The authors should give some info on how many subjects get actually treated with long course primaquine in the region; low access to primaquine could completely justify the lack of haemolytic reactions.**

No reliable data on number of PQ doses provided are readily available. In order to address the comment we have changed the respective sentence, it now reads: “*The reasons for this are unclear, but could reflect low prescription of PQ by healthcare providers, underreporting of adverse events due to limited access to healthcare, poor treatment adherence to a prolonged course of PQ, or G6PDd individuals having partial protection against malaria (15, 44, 45)*.” (lines 334 – 337)

24. **Line 331 if it is not significant, it is not higher! I also do not understand this sentence “malaria status was not found to be a significant predictor of G6PD activity irrespective of diagnostic assay applied.”**

G6PD activity was higher among patients with microscopic malaria. The difference did not reach statistical significant possibly because numbers were small and the study was underpowered. The following sentence reflects this: *“While not statistically significant, the median G6PD activity was almost 25% higher (1.7U/gHb) among patients with malaria diagnosed by microscopy or RDT compared to participants with a negative result.”* (lines 339 – 341).

25. **Line 335 how many papers instead do report a good correlation between G6PD genotype and phenotypes?**

We have rephased this sentence which now reads: *“Although G6PD genotype is a key determinant of enzyme activity (46), we observed poor correlation between G6PD genotype and phenotype , a phenomenon that has been described previously (47-50).”* (lines 344 - 345)

26. **Figure1 shows that the distribution in males, although bimodal, has a large number of subjects in the intermediate range**

Since the study population is multi-ethnic with different genotypes present in the population, males hemizygous for a mild G6PD variant may be classified as intermediate G6PD activity within the entire population when applying a population wide AMM

27. **Figure 2 - I would show only males**

Figure 2 shows the distribution of G6PD activity per ethnicity. We believe that this should include females to remain consistent with the overall aim of this paper, which was to assess the use of routine G6PD testing to guide primaquine based radical cure in multi-ethnic populations.
