# Supplementary material for: Wide range of G6PD activities found among ethnic groups of the Chittagong Hill Tracts, Bangladesh
Source: PLoS Negl Trop Dis. 2020 Sep 14;14(9):e0008697. doi: 10.1371/journal.pntd.0008697 (PMC7514097; doi:10.1371/journal.pntd.0008697)
Supplement: Attachment — Submitted filename: Ley et al_PLoSNTD_Response to Reviewers_2_v4.docx [file pntd.0008697.s008.docx]

Dr Wuelton Monteiro

Associated Editor

*PLoS NTD*

6^th^ August, 2020

**Re.** PNTD-D-20-00793

*"Wide range of G6PD activities found among ethnic groups of the Chittagong Hill Tracts, Bangladesh"*

Dear Dr Monteiro,

Thank you for your email regarding our manuscript and the opportunity to respond to further comments. Reviewer #1 and #2 agree with the provided changes, reviewer #3 has further queries which are very similar to the ones stated previously.

We have now responded to these below. We have also identified a typo in the supplementary table 3 that describes parameters of the regression analysis. The adjusted R2 should be 0.5513 rather than 0.5340 on line 545.

We have reviewed the entire manuscript and discussed the analysis with a biostatistician to address some of the statistical issues regarding the Hb measurements as well as the use of non-normalized G6PD activities for our models below.

We also received feedback following resubmission of the manuscript from the editorial team and have included our responses in this replay as well.

We hope that you would agree that the paper is now suitable for publication.

Thank you for your time and consideration.

Yours sincerely,

Benedikt Ley on behalf of the other authors

**Editorial team:**

**We note that we have 2 Supporting Information files and 0 Supporting Information legends. Please ensure that all Supporting Information files are included correctly and that each one has a legend listed in the manuscript.**

Supporting information legends are placed at the end of the manuscript. We have added a legend for the underlying database (line 547)

**Please clarify whether or not a parent or guardian provided consent on behalf of the minors to participate in the survey.**

We have clarified this further, the respective sentence now reads (lines 220 – 222): “*Written informed consent was collected from all participants or their legal guardians prior to enrolment and in addition written assent was collected from all minors above the age of 11 years.”*

**Funding details:**

Funding details are included from line 380 onwards. We have streamlined the text, which now reads (lines 380 – 387): “*This study was funded by Bill and Melinda Gates Foundation (OPP1054404 and OPP1164105), and the Eck Institute for Global Health at the University of Notre Dame. BL is funded by the Australian Department of Foreign Affairs and Trade, RNP is funded by the Wellcome Trust (Senior Fellowship in Clinical Science, 200909), KT is a CSL Centenary Fellow. SA is supported by the Bill and Melinda Gates Foundation (OPP1054404), Wellcome Trust (200909 awarded to RNP) and a Georgina Sweet Award for Women in Quantitative Biomedical Science.*

*No funding bodies had any role in study design, data collection and analysis, decision to publish, or preparation of the manuscript.”*

**Reviewer #3:** **The authors have adequately addressed some of my questions but there are a number of issues that have not been resolved and some have arisen during revision. In general, I feel the authors have not explored all the aspects of the questions and comments received from the reviewers.

They state their main aim in line 34 and again line 102: “Aim of this study was to determine the need for routine testing within a multi-ethnic population by assessing the prevalence of G6PDd among the local population”. If the aim was to assess the prevalence of G6PD deficiency, a qualitative test (in the field or in the lab) would have been sufficient. The authors took a brave decision to perform a rather more complicated work in order to bring more details and possibly useful data but then fail to perform the reference test in the correct way (using Hb from previous assessment) and in their main statistical analysis they do not use the right values of G6PD activity (see my comment below). After showing that all ethnic groups have a moderate to high prevalence of G6PD deficiency they reach the conclusion that “practically” G6PD screening might still be done based on ethnicity (even after 3 reviewers suggested that was not the right interpretation).**

We thank the reviewer for the additional comments. A qualitative test discriminates between G6PD activities below and above 30% enzyme activity, but does not quantify individuals with intermediate deficiency. In view of the apparent risks of haemolysis in patients with intermediate deficiency and the restriction of tafenoquine to patients with greater than 70% activity, quantitative testing is becoming increasingly relevant. Hence our analysis used data from quantitative spectrophotometry to provide greater information in our investigation of the Chittagong Hill Tracts (CHT) populations.

In response to the reviewers comment on the concluding remarks regarding stratification of risk according to ethnicity, we agree that this could be worded and better and have revised the discussion again as follows (lines 366 – 374):

*“In conclusion, there was a high prevalence of G6PDd in the CHT, but this varied significantly between ethnic groups. Whilst testing should ideally be made widely available to identify individuals with G6PDd, in practice this is often unavailable or unreliable due to financial and logistical constraints. Priority should be given to ensuring the availability of G6PDd testing in communities where the prevalence of G6PDd and thus hemolytic risk is greatest. In areas where testing is not available, consideration should be given to administering primaquine with additional measures to mitigate severe adverse reactions by detecting early signs of hemolysis. Determining the prevalence and variants of G6PDd in malaria endemic areas with different ethnic groups will inform public health interventions to ensure that primaquine radical cure can be provided safely and effectively. ”*

**And while it is useful to know that the laboratory performing the analysis collaborates with WHO and PATH, I believe the comments and suggestions from reviewers where certainly not about the quality or reputation of the laboratory but about the quality of data presented, possibly the study design and quite clearly the conclusions.**

We agree that laboratory quantification of G6PD activity can be problematic and state as such in our conclusions. However we seek to reassure that appropriate quality control measures were in place at icddr,b and that the reference laboratory operates according to globally accepted standards.

**Below there are few more comments to specific issues:**

**1) In my first review I had asked about the accuracy of Randox G6PD assessment and to specify whether testing was carried out in single replicate or duplicate. Accuracy is not reported and there is no mention of duplicates in the methods but there is a mention in line 359-361 of discussion. This should be written in the methods.**

We have added the following sentence to the methods section *(lines 153 – 155):*

*“All samples were measured in duplicate, and a third measurement was performed if the measurements differed by more than 10% of the upper value.”*

**It is also unclear to me why the haemoglobin was not re-assessed at the laboratory when the samples were analysed for G6PD, which is in my opinion one of the biggest technical limitations of this study. The authors replied that in previous studies they have tested samples kept in laboratory for up to 1 week but I presume they had retested both G6PD and Hb over time.**

We have previously assessed the decay of G6PD activity over time at 4°C (Alam et al, PLoS One 2018, DOI: 10.1371/journal.pone.0206331). When considering G6PD activity normalized by Hb, any significant change in Hb over time would have altered G6PD activity. However, there was no change in normalised G6PD activity over the course of seven days. In the current study the median time to testing was two days (IQR: 1-4, range 0-7), we thus believe that the Hb measurement collected in the field are robust.

We have added the respective reference, the corresponding text now reads (lines 361 – 363):

*“However, all samples were tested within seven days, the recommended maximum interval between venepuncture and measurement of G6PD activity (33).”*

We have also clarified this in the methods section, where it now reads (lines 149 – 150):

*“Since samples can hemolyze during transport, G6PD activity was only measured if there were no visible signs of hemolysis.”.*

**2) In the multivariate analysis, the variable listed in the methods (line 199-202) are not the same as those listed in the results (315-317). For example, “ethnicity” is missing in the variable listed in methods.**

We have revised the test in the methods to include “ethnicity” as independent variable (line 204).

**And on a more substantial aspect of this analysis:
a) I think the authors are wrong in using the “non-normalized” values of absorbance, because G6PD activity is defined as normalized by either Hb or number of RBCs. The “non-normalized” absorbance is simply not the enzymatic activity.**

We agree that G6PD activity must be normalized in order to be meaningful. Hence in the multivariable models even though the G6PD activity was non-normalised as the dependent variable, Hb was included an independent variable and thus this ensures appropriate correction. We have revised the text on lines 200 – 202 to clarify:

*“Non-normalized G6PD activity (in U/dL) was used as the dependent variable and Hb concentration included as a covariate.”*

**b) While they make a point of not using Hb-normalized G6PD in order to then analyse Hb as independent variable, they use a lot of variables that are actually collinear (sex and heterozygote genotype, sex and weight, sex and height) and others that might be collinear (for example if a mono-ethnic village was very far away and samples analysis was delayed, then “ethnicity” and “delay to test” variables would be collinear). I believe this should be checked and made clear in the analysis.**

We discussed the analysis again with a biostatistician and reviewed the model for co-linearity by calculating the variance inflation factor (VIF). All variables in the initial and final model were well below the threshold for multi-co-linearity. We have added this to the manuscript in the methods (lines 207 – 208):

*“Models were assessed for multi-co-linearity by calculating the variation inflation factor (VIF), with co-linearity defined when VIF was >10.”*

We have also added this in the results section accordingly (line 321):

*“**There was no significant multi-co-linearity in either the initial or final models.”*

**3) Lushai ethnic group: a sentence in the text would be enough to report that G6PD deficiency was found in 1 person of this ethnicity, without needing to add this ethnic group in the table.**

Following the same comment in the last review we had previously set “Lushai” in italics in tables 1 and 2 to distinguish these participants and have removed Lushai from figure 3.

Since we collected blood form these two participants and analysed the corresponding samples, we would like to present these data as well, not least since these are the first reported G6PD activities ever from this ethnicity.

**4) The authors confirm that “Although G6PD genotype is a key determinant of enzyme activity, we observed poor correlation between G6PD genotype and phenotype, a phenomenon that has been described previously (44-47).” The first paper used retrospective hospital data collected during a decade (1993-2003) and phenotype were defined using four different ranges over time; hardly comparable to the current study. The second paper shows similar poor genotype-phenotype correlation and indeed used the same reagents as the current one. I think there are many reasons to believe that the poor correlation observed here and rarely in other settings is due primarily to technical issues rather than reflecting a true biological phenomenon.**

The cited references provide evidence of the wide variability of G6PD measures even within homozygous, hemizygous and heterozygous individuals (ie low correlation between phenotype and genotype). In his recent review of G6PD deficiency Luzzatto et al also suggests that G6PD readings may be affected by recent haemolytic events (Luzzatto et al (2020) in Blood, DOI: 10.1182/blood.2019000944). We agree with the reviewers that the observed variation may be due host and environment factors as well as assay variability as described by Pfeffer et al (2020, PLoS Med, DOI: 10.1371/journal.pmed.1003084). We have added both references and changed the text accordingly (lines 345 - 347):

*“Although G6PD genotype is a key determinant of enzyme activity (43), we observed poor correlation between G6PD genotype and phenotype, a phenomenon that may reflect both assay variability as well as host and environmental factors (44-49).”*
